# Assessing the Role of Incubation Temperature as a Barrier to Successful Establishment of Coho Salmon ( *Oncorhynchus kisutch* ) in a Rapidly Warming Arctic

**DOI:** 10.1002/ece3.70797

**Published:** 2025-01-11

**Authors:** Elizabeth D. Lindley, Karen M. Dunmall, Peter A. H. Westley

**Affiliations:** ^1^ College of Fisheries and Ocean Sciences University of Alaska Fairbanks Fairbanks Alaska USA; ^2^ Freshwater Institute Fisheries and Oceans Canada Winnipeg Manitoba Canada

**Keywords:** Arctic, climate change, coho salmon, embryonic development, hatching

## Abstract

Warming associated with climate change is driving poleward shifts in the marine habitat of anadromous Pacific salmon (*Oncorhynchus* spp.). Yet the spawning locations for salmon to establish self‐sustaining populations and the consequences for the ecosystem if they should do so are unclear. Here, we explore the role of temperature‐dependent incubation survival and developmental phenology of coho salmon ( *Oncorhynchus kisutch* ) as a potential early life history barrier to establishment in an Arctic stream. We exposed embryos to temperatures previously recorded in the substrate of an Arctic groundwater spring‐fed spawning environment. Using a common garden experimental design, coho salmon embryos were exposed to treatments that thermally mimicked four spawning dates from August 1 to October 1 (AUG1, SEPT1, SEPT15, and OCT1). Spawning temperatures were 6°C at the warmest (AUG1) and 1.25°C at the coldest (OCT1). We observed low survival rates in SEPT1 (41%) and OCT1 (34%) and near complete mortality in the other treatments. While far below what is considered normal in benign hatchery‐like conditions, these rates suggest that temperatures experienced at these spawning dates are survivable. We detected differences in developmental rates across treatments; embryos developed 1.9 times faster in the warmest treatment (AUG1, 120 days) compared to the coldest (OCT1, 231 days). Differences in accumulated thermal units (ATUs) needed for hatching ranged from 392 ATUs in AUG1 to 270 ATUs in OCT1, revealing compensation in developmental requirements. Given these findings, the most thermally suitable spawning dates within our study are between September 15 and October 1, which facilitates hatching and projected nest emergence to occur in spring warming conditions (March–September). Broadly, our findings suggest that spawning sites within thermal tolerances that can support the survival and development of coho salmon exist in the North American Arctic. Whether the habitat is otherwise suitable for transitions through other life stages remains unknown.

## Graphical abstract

We explore the role of temperature‐dependent incubation survival and phenology of coho salmon ( *O. kisutch* ) as a potential early life history barrier to establishment in Arctic streams. Our results suggest that spawning sites within thermal tolerances that can support the survival and development of coho salmon exist in the North American Arctic.

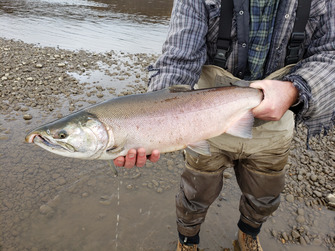

## 1 Introduction

Aquatic and terrestrial Arctic borealization is one of the strongest signals of warming global temperatures and is transforming patterns of biodiversity and ecosystem functioning (IPCC 2014). Mueter et al. (2021) summarized the likely biological responses by marine organisms to projected physical changes in the Arctic, including: regional enhancement in Arctic waters, reduced production in subarctic waters, phenological mismatch and poor recruitment, northward shifts, and cascading food web effects. Consistent with these hypothesized responses, increasing observations of poleward‐bound native and non‐native species have the potential to compound pressure on recipient ecosystems already experiencing abrupt physical perturbations (Melbourne‐Thomas et al. 2022). While habitat expansion may allow for pathways of resilience in a changing world, climate‐mediated movement of marine species into the Arctic has potential to restructure ecosystems, and thus impact food security, cultures, and societies (Frainer et al. 2017; Pinsky et al. 2018; Chila et al. 2022). Exploring dynamics of potential establishment by redistributing species is imperative, as ephemeral encounters with potentially minimal impacts can trend towards organisms becoming long‐standing fixtures with permanent impacts.

**FIGURE 1:**
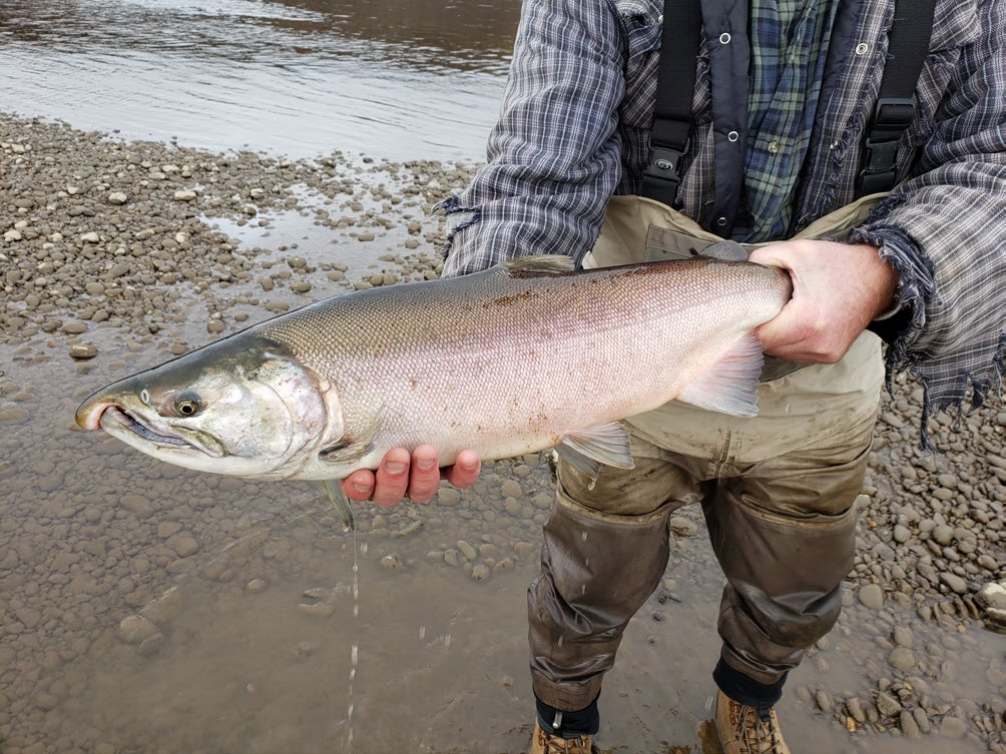
Coho salmon ( *Oncorhynchus kisutch* ) caught on the Itkillik River, Alaska. 2018. Photo by B. Scanlon, Alaska Department of Fish and Game.

In recent years, all five species of semelparous Pacific salmon (*Oncorhynchus* spp., hereafter referred to as “salmon”) are thought to be shifting in their North American Arctic relative abundance and range (Dunmall et al. 2013, 2021). On the North Slope of Alaska, all five species of salmon have been increasingly encountered in subsistence fisheries (George, Moulton, and Johnson 2009; Mikow et al. 2016; Carothers et al. 2019). However, the shifts of salmon in Arctic Alaska are nuanced, where some species such as pink and chum salmon have a long‐standing presence in the region (Craig and Haldorson 1986; Nielsen, Ruggerone, and Zimmerman 2013), and others have been seldom seen until more recently (Carothers et al. 2019). Chinook ( *O. tshawytscha* ), sockeye ( *O. nerka* ), and coho salmon ( *Oncorhynchus kisutch* ) are historically rarely encountered across the North Slope, with the latter being the rarest, having only a few formally documented encounters (Craig and Haldorson 1986; Nielsen, Ruggerone, and Zimmerman 2013). In 2018, a novel encounter with a coho salmon was documented in the Itkillik River (Figure 1; B. Scanlon, pers. comm., April 30, 2021), a putative salmon‐supporting tributary of the North Slope draining Colville River (Carothers et al. 2019; Giefer and Graziano 2023). These observed changes in salmon on the North Slope suggest deteriorating thermal barriers to accessing the Arctic (Farley et al. 2020; Dunmall et al. 2024). Overall, the observed shifts in the distribution and relative abundance of salmon across the North Slope exemplify the complexities in physiology and differences in life history across species, and how species‐specific shifts may scale in response to a changing climate.

Perceived shifts in salmon abundance and species compositions in North Slope subsistence fisheries raise questions regarding their spawning activity and potential interactions with culturally important subsistence species, such as Dolly Varden ( *Salvelinus malma* ), Arctic cisco ( *Coregonus autumnalis* ), and broad whitefish ( *C. nasus* ) (Brown et al. 2016; Carothers et al. 2019; Chila et al. 2022). One driver for concern is that Arctic spawning salmon have the potential to overlap in spawning timing, and more importantly, utilize similar spawning habitat characteristics to those of Dolly Varden and Arctic char ( *S. alpinus* ) in cold Arctic systems (Dunmall et al. 2016; Bilous and Dunmall 2020)). Lending support for this concern, a juvenile chum salmon captured near Kaktovik, Alaska shifts the biologically confirmed spawning distribution of chum salmon in the Arctic (Dunmall et al. 2022). Thermal refugia in the form of groundwater springs in Arctic systems provide relative thermal stability (Meisner, Rosenfeld, and Regier 1988; Power, Brown, and Imhof 1999), creating oases of suitable incubating conditions for cold‐adapted fall spawners, such as chars, and potentially salmon (Dunmall et al. 2016). Indeed, the true barrier for establishment of new salmon populations may lie within the immobile early life history of these fishes (Dunmall et al. 2016), and groundwater‐fed springs are more likely to support establishing populations of salmon, as liquid water remains year‐round when other sites within these cold systems freeze solid from the surface to the substrate (Power, Brown, and Imhof 1999). While chars and salmon are known to co‐exist in other systems that support salmon across Alaska, the impacts of establishing populations (e.g., on the North Slope) are unknown. An improved understanding of the establishment of salmon in Arctic Alaska is critical, particularly when considering potential interactions with local fishes.

The effect of temperature on embryonic survival and development of salmon has been long studied in laboratories and is most confidently understood under constant incubation temperatures (Bailey and Evans 1971; Murray and Beacham 1986). Few studies have considered realistic thermal profiles (Steel et al. 2012), and even fewer have considered embryonic survival at temperatures approaching lower thermal thresholds (Bailey and Evans 1971; Tang, Bryant, and Brannon 1987). Survival during early incubation declines dramatically in temperatures at and below 4°C, with especially critical thermal sensitivity in the very initial stage of development when the embryo is less than 128 cells (Combs 1965; Beacham and Murray 1987; Tang, Bryant, and Brannon 1987). Keeping other variables stable, salmon embryo development in colder temperatures requires more time to reach a similar stage to those reared at “normal” or warmer temperatures. However, this relationship is not linear, as embryos in low temperatures are observed to require fewer accumulated temperature units (ATU) to hatch compared to counterparts incubating at higher temperatures (Brannon 1987; Quinn 2018). This developmental compensation may be the key to successful Arctic spawning, as it shrinks the hatching window and can minimize mismatches between spawning time and developmental requirements (Brannon 1987; Quinn 2018). Of the Pacific salmon species, coho salmon have the lowest average thermal tolerance during incubation with 90% survival as low as 1.3°C (Tang, Bryant, and Brannon 1987), and the most rapid embryonic development compared to other Pacific salmon species at all temperatures (Beacham and Murray 1990). These unique characteristics, considered alongside the capacity for developmental compensation during incubation in response to cold temperatures contribute to the thermal likelihood for successful establishment in the Arctic.

The goal of this study was to explore the potential for coho salmon to successfully incubate in an emulated Arctic spawning thermal environment at different spawning times. Specifically, our objectives were to (1) quantify survival within and among emulated spawning dates ranging from August 1 to October 1 in temperatures recorded in an Arctic groundwater spring, and to (2) quantify developmental rates and timing in four emulated spawning dates. We report the results of a common garden rearing experiment tooled to address our objectives. Given the thermal principals of incubation, we expected that the earlier spawning may support greater rates of incubatory survival and more rapid development contrasted by longer development and lower rates of survives in later spawning dates.

## 2 Methods

### 2.1 Study Population and Experimental Animals

We used a population of coho salmon that typically spawns in the first 2 weeks of October from Ship Creek, a watershed draining into Cook Inlet in Southcentral Alaska. On October 13, 2021, gametes from 10 ripe females and 20 ripe males were stripped and contained in labeled individual Whirl‐pak sampling bags and immediately placed in a chilled cooler. Stream temperatures at the time of egg takes was 4.5°C. Gametes were then immediately transported 577 km from the sampling site to the laboratory site at the University of Alaska Fairbanks. Fertilization took place in the rearing laboratory at the Biological Research Diagnostic Facility at the University Alaska Fairbanks. Eggs from females were crossed with milt from two males creating a total of 10 half‐sib families, following procedures of Sparks et al. (2017). Fertilized eggs were then allowed to water harden for 10 min. This half‐sibling family design increased the potential for fertilization success and generally emulates breeding systems observed in nature, where multiple males will fertilize a single clutch of eggs (Esteve 2005).

### 2.2 Thermal Laboratory Experiments

Following water hardening, embryos of each family were placed into vertical incubators and exposed to four temperature treatments (see details in next section). Vertical incubators were supplied with a recirculating supply of 375 L of water, at an average flow of 18 L/s. Each treatment received four replicates per family, and each replicate was housed in a separate tray within the incubator (termed T1–T4). Ova were held in PVC cups measuring 7.62 cm diameter × 6.35 cm tall with a screened bottom. Each of the four replicates held approximately 55 fertilized ova and were haphazardly placed into each tray level of the vertical incubator to control for any subtle variation in temperature changes as water flowed through the apparatus. Two environmental chambers housed two treatments each and chambers were held at constant temperatures and maintained in constant darkness except during sampling, when red light was used.

### 2.3 Temperature Treatments

**FIGURE 2:**
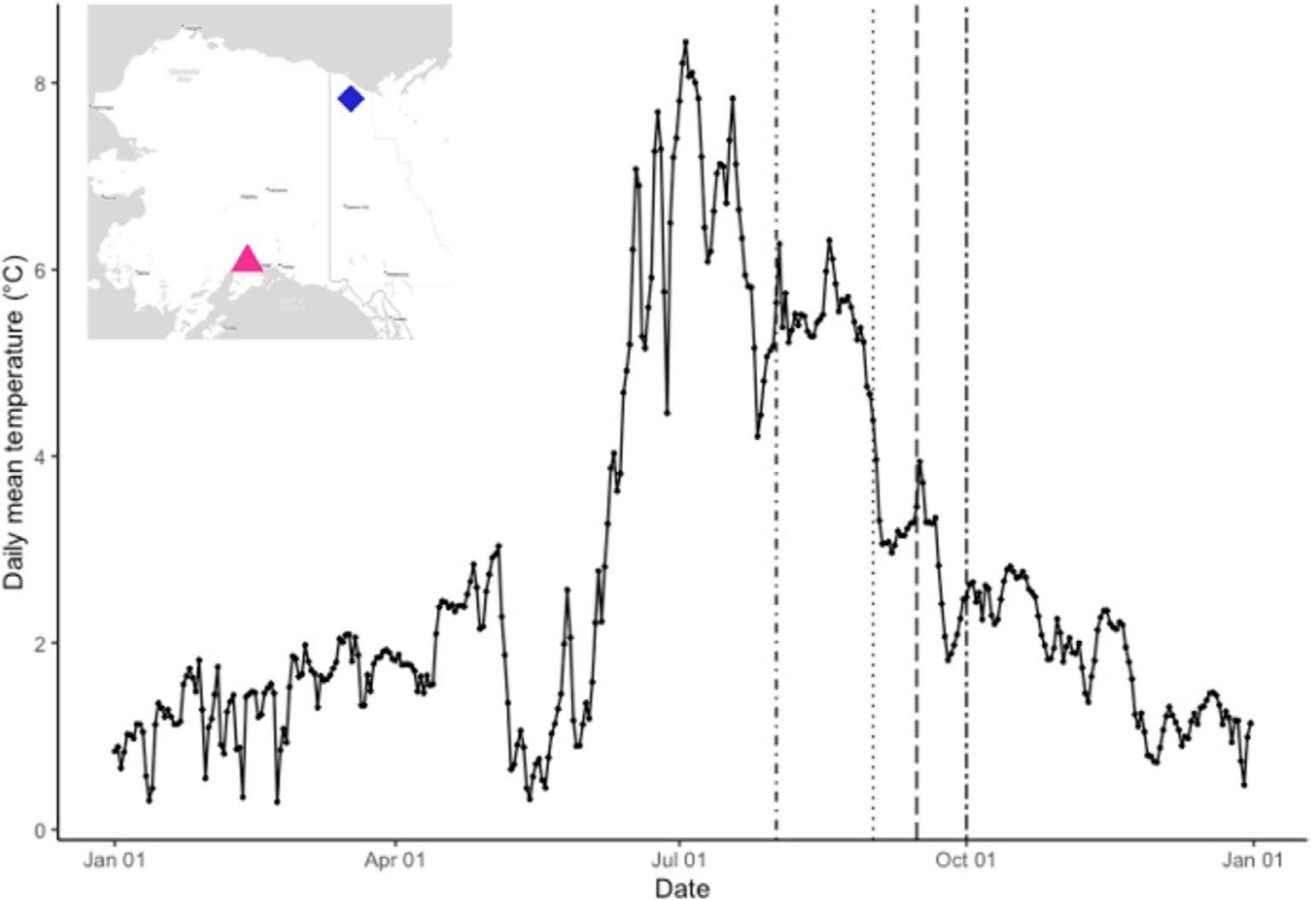
Thermal profile of stream substrate recorded in the Babbage River, Yukon Territories, Canada (68.6*°* N, −138.7*°* W; collected during 2013 as reported in Dunmall et al. 2016), overlain with experimental spawning dates (dashed lines) used in four thermal treatments used to rear Alaska coho salmon ( *O. kisutch* ) sourced from Ship Creek, Alaska. The inset map identifies the locations of the Babbage River (blue diamond) and Ship Creek, Alaska donor population (pink triangle, map made using ggmap package in program R).

**FIGURE 3:**
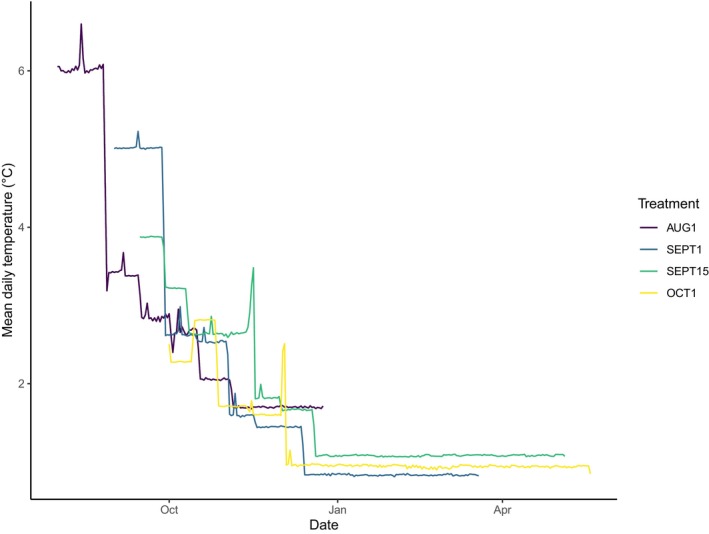
Thermal profiles of experimental treatments used for rearing coho salmon (*O. kistuch*) embryos in a laboratory setting. Each treatment emulates spawning dates and mimics subsequent thermal delivery as recorded in a groundwater spring in the Babbage River, Yukon Territories, Canada. Each treatment profile concludes once all embryos within that treatment hatched.

We exposed developing embryos to four temperature treatments that emulated spawning dates based on timing of observations of salmon encounters in North Slope rivers and from biological first principles of the species (Bendock 1981; B. Scanlon, pers. comm., May 3, 2023). We applied realistic temperatures for spawning and incubation that were documented at a Dolly Varden spawning site in an Arctic river (Babbage River; 68.8° N, −138.7° W), a location that is associated with a perennial groundwater spring that provides a thermal refugia for fish during winter (Figure 2; Dunmall et al. 2016). Each treatment was initiated at progressively later spawning dates and followed natural variability in thermal delivery to the nearest 0.5°C, emulating our experimental stream (Figure 3). Specifically, the four treatments were: (1) AUG1, initiated or spawning at 6.0°C, (2) SEPT1, initiated at 5.0°C, (3) SEPT15, initiated at 3.8°C and (4) OCT1, initiated at 2.6°C. We will refer to our treatments as spawning dates hereafter. Each progressively later spawning date exposed embryo to both cooler temperatures during ‘spawning,’ including the known post‐spawning critical thermal window, and a lower average thermal exposure over the course of incubation. A deviation in our planned approach can be seen in SEPT15 and OCT1, where a loss in power to the facility resulted in our chillers to shut off and environmental chambers to warm (Figure 3).

Experimental temperatures were recorded with a HOBO Water Temp Pro v2 logger (15‐min intervals, accurate to ±0.2°C) placed in the bottom tray of each incubator. A hand thermometer was used to measure temperatures in trays and holding tank during daily checks. Temperatures were adjusted each day as needed to match daily temperature profile for each treatment. Dead embryos were removed from treatments the day after original placement in experimental apparatuses as they were considered transportation mortalities. In contrast, all dead embryos thereafter were removed on a daily to biweekly basis and used in analysis. To avoid any physical disturbances, entrance into to incubation chambers during the known period of mechanical sensitivity, 80–120 ATUs (Quinn 2018), was prohibited.

Hatching larvae (termed alevins) were removed and sacrificed in an overdose of MS‐222 on the day they hatched. Each day of sampling, alevins were weighed to the nearest 0.001 g. Only the first 25 individuals from each family in each treatment were weighed, given minimal within‐family variation in size (Sparks et al. 2017). All live animal activities detailed were reviewed and approved by UAF Institutional Animal Care and Use Committee protocol #1758945‐2 and Alaska Department of Fish and Game Aquatic Resource Permit #P‐21‐018.

### 2.4 Analysis

We fitted a generalized linear model (GLM) and a linear mixed‐effects model (LMM; Bolker et al. 2009) to examine the effects of spawning date on survival and developmental rates, respectively. Both models included temperature treatment and incubator tray level as fixed effects. The LMM also included family identity nested within spawning date as a random variable. The GLM was fitted with a binomial distribution (i.e., 0 represented embryo that died, and 1 embryo that survived to hatching) and a binomial logit link. All analyses were done in Program R (R Core Team 2022) and mixed models were analyzed in package lme4 (Bates et al. 2015). All visualizations were created using the “ggplot2” package in R (Wickham 2016).

We used a well‐established predictive model developed from observations in experimental rearing of all five species of Pacific salmon (Beacham and Murray 1990) to predict day to hatch for each of our treatments and compared these with observed data.

## 3 Results

### 3.1 Embryo Survival

**FIGURE 4:**
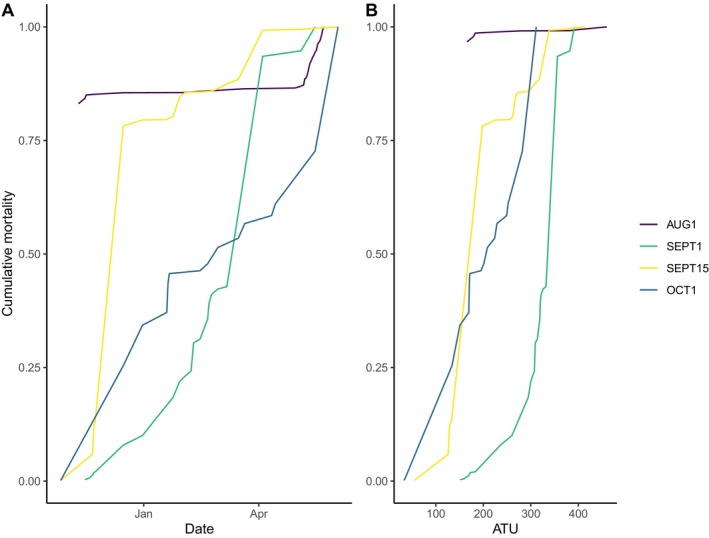
Cumulative mortality of coho salmon ( *O. kisutch* ) embryos reared in a Fairbanks, Alaska laboratory and exposed to four treatments mimicking spawning dates within an Arctic groundwater stream in the Babbage River, Yukon Territories, Canada. Panel (A) represents cumulative mortality over time, and panel (B) represents cumulative mortality at developmental stage as measured through accumulated thermal units (ATUs).

Embryo survival responses yielded uncertain data. Survival was unexpectedly low in two dates, AUG1 (8.3% survival) and SEPT15 (2.8% survival)—rates that approached complete mortality and occurred for unknown reasons (Figure 4). These dates contrasted SEPT1 (41.3%) and OCT1 (34.6%) which had markedly higher, albeit still modest, rates of survival (Table 1). Analysis of this equivocal data using Equation 1 revealed that AUG1 (*p*‐value = 0.0007), SEPT1 (*p*‐value = 0.002), and OCT1 (*p*‐value = 0.03) had a significant effect on our observed survival rates. SEPT15 (*p*‐value = 0.28), did not significantly affect our observed survival rates thus we are very hesitant to attribute our observations of survival to the temperature treatment itself. The fixed effect of tray level also had some significant contribution to our observed survival rates (*p*‐values < 0.0001 for T1–T4), meaning that survival rates slightly differed among trays. T2–T4 consistently had higher survival than T1 (log likelihood estimates: T2 = 1.1, T3 = 2.2, T4 = 1.9); however, there was no pattern to suggest that each lower tray had higher survival than the one above it.

**TABLE 1:** Summary of environmental conditions and observed and predicted phenological responses from a coho salmon ( *O. kisutch* ) rearing experiment using incubatory temperature treatments that emulate an Arctic spawning location.

Treatment	Mean temp (°C)	Temp SD	Median days to hatch	Predicted days to hatch^a^	Median ATU to hatch	Calendar median hatch day	Predicted emergence^b^	Survival (%)
AUG1	2.98	1.50	120	106	392	November 29	March 16	8.3
SEPT1	1.85	1.40	161	152	362	February 9	April 13	41.3
SEPT15	1.70	0.89	188	165	353	March 21	April 27	2.8
OCT1	1.25	0.55	231	174	270	May 20	September 9	34.6

^a^ Predictions calculated using a hatch estimation model from Beacham and Murray (1990).

^b^ Prediction rounded to nearest 0.5°C using generalized estimations from Beacham and Murray (1990) and Quinn (2018).

### 3.2 Hatching Timing

**FIGURE 5:**
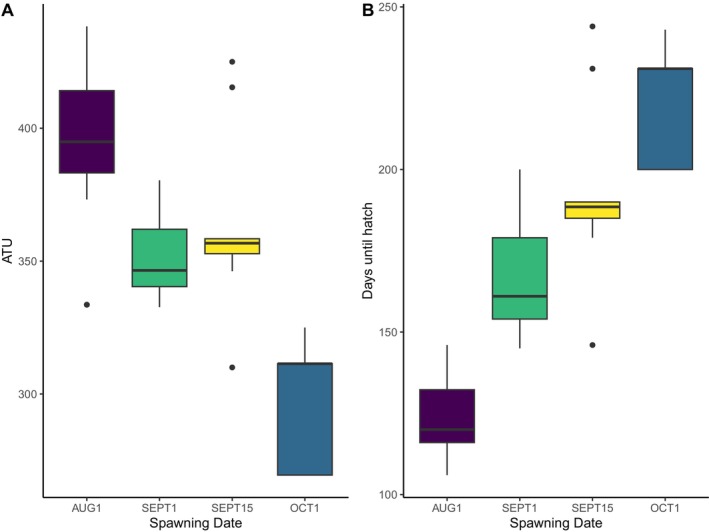
Box plots representing developmental rates of incubating coho salmon ( *O. kisutch* ) reared in Fairbanks, Alaska at four experimental spawning dates that emulate stream conditions in an Arctic river. Panel (A) represents accumulated thermal units (ATUs) needed to hatch, and panel (B) shows days until hatching. Treatments are arranged from earliest (warmest) to latest (coolest) spawning dates.

Developmental rates, as measured by the number of ATUs needed to hatch, were a function of temperature (Figure 5). Consistent with our expectations, all families developed most rapidly in the warmest and earliest spawning day (AUG1, average exposure until all embryo hatched = 2.98°C), with substantial decreases in later spawning dates (Table 1). The average developmental requirements to hatch in the earliest and warmest spawning date (AUG1) was 124 days (SD = 11.8) and 398 ATUs (SD = 22.9). In contrast, the latest and coldest spawning date (OCT1), required 219 days (SD = 16.7) or 294 ATUs (SD = 22.1). This is a difference of 104 ATUs or 95 days required for an egg of the same source to hatch. Our model detected significant effects for all spawning dates but one, SEPT15 (*p*‐value = 0.051). AUG1 (*p*‐value = < 0.01), SEPT1 (*p*‐value = < 0.01), and OCT1 (*p*‐value = < 0.01) all significantly explained observed developmental rates. In other words, spawning dates significantly affected how quickly embryos developed and how many ATUs were required to reach hatching for three of our four treatments. The two treatments with low survival also had less variance in days to hatching when compared to the treatments with higher survival (Figure 5). We also detected evidence for variability in development rates across families within each spawning date with variance values AUG1 = 96 ATUs (SD = 9.81), SEPT1 = 114 ATUs (SD = 10.7), SEPT15 = 690 ATUs (SD = 26.27), and OCT1 = 270 ATUs (SD = 16.43). Similar to our observations in survival, tray levels also had some statistically significant differences in developmental requirements (*p*‐value < 0.05), where trays 2–4 required more ATUs than tray 1 (T2 = 8.1 ATUs [SD = 2.4], T3 = 9.7 ATUs [SD = 2.4], T4 = 4.8 ATUs [SD = 2.4]), perhaps due to slight differences in temperature as water flowed from top (T1) to bottom (T4). The variance across the random effect of family intercepts was 96 ATU (SD = 9). We also detected some within‐treatment variability in developmental rates, where colder spawning dates seemed to have greater variability in ATU requirements than warmer spawning dates (Figure 5).

## 4 Discussion

Within the context of Arctic spawning by coho salmon, this study suggests that spawning conditions within Arctic thermal refugia can support embryonic survival and incubation of potentially establishing subarctic coho salmon. Consistent with our expectations, we detected significant effects of spawning date on developmental rates, with provisional interpretation of a similar effect on survival. The unexpectedly low survival in AUG1 and SEPT15 may be artifacts at best or be influenced by factors that potentially also impacted SEPT1 and OCT1 to an unknown extent (e.g., mechanical disturbance, light exposure, chemical contaminants, equipment cleanliness, etc.). Compensation in development between the warmest and coldest spawn dates was 104 ATUs, which was an observed difference of 95 days in our experimental conditions. While the use of *average* thermal differences oversimplifies the developmental relationship, this demonstrates a compensatory hatching window of nearly 3 months driven by thermal variability within a mere 1.7°C. This means that, within an average difference of 1.7°C, emulated spawning dates were able to compensate for hatching requirements within a 3‐month timeframe. There was considerable variability in development across families, and to a lesser degree in survival. Taken as a whole, our findings suggest that there are phenological opportunities for early life‐history stages to successfully develop by coho salmon that spawn in Arctic groundwater‐fed oases.

Survival varied among spawning dates, with some observations counter to our expectations. AUG1 and SEPT15 treatments had survival rates nearing complete mortality for unknown reasons, which makes any conclusions regarding survival in our study provisional at best. Despite low survival, AUG1 maintained significance in our analysis; however, we cannot logically attribute these survival results to the temperature exposure. Due to our inability to attribute the anomalous results in AUG1 and SEPT15 to any single environmental condition or developmental disturbance, we must consider that survival rates in SEPT1 and OCT1 may have also been affected to an unknown degree. As such, we exercise caution in the treatment of our data and cannot draw any strong conclusions regarding the effects of temperature on survival. However, within the treatments that may allude to more realistic survival responses (SEPT1 & OCT1), we saw decreased survival from the warmest (41.3%) to the coldest spawn date (34.6%), consistent with our expectations. We must acknowledge that survival rates are quite low for a laboratory experiment and that the 7% difference between these two treatments have equivocal biological interpretations. Nonetheless, the 7% difference does scale in the direction we anticipated. Existing studies use the term “normal” for high survival levels in experimentation, often exceeding 80% (Combs 1965; Bailey and Evans 1971). While perhaps our results fall far from what is considered normal survival in experimentation, we can say that the temperatures experienced by embryos spawned on September 1 and October 1 in our emulated system are survivable by coho salmon. However, we can only speculate to how these results translate to the natural conditions of the Arctic.

The geographic salmon landscape follows a general trend that cold‐spawning populations (i.e., high vs. low latitude populations) spawn earlier to allow for a match between ATU requirements and alignment with peak periods of resource availability (Einum and Fleming 2000; Quinn 2018; Armstrong et al. 2021). This wide spawning window is also a stabilizing tactic that can act as a buffer against environmental disturbances or other forms of spawning intercepts (Hilborn et al. 2003). Therefore, it might be expected that coho salmon potentially establishing in Arctic streams may spawn early in the fall to ensure that enough thermal units can be accumulated during the long cold winter. Counter to this expectation, we found that it might be beneficial for Arctic establishing coho salmon to spawn later given observed developmental compensation and an extended target hatch and emergence. Within a mere 2 month spawning window and 1.7°C in average thermal conditions, compensation in required ATUs to achieve hatching between the earliest and latest spawning was 68 ATUs (median), and an observed 111 days. In other words, embryos spawned on August 1 required 68 more ATUs to hatch compared to those spawned on October 1, which took 111 days to accumulate. The use of average temperature exposure in our experiment, however, oversimplifies the thermal delivery (i.e., thermal variability or constancy) and does not capture the quick accumulation of thermal units just after spawning, such as was experienced by AUG1 embryos– an important phenomenon in cold Arctic spawning fishes (Dunmall et al. 2016).

The level of compensation for the same clutch of embryos reared in slightly different thermal environments would allow for 50% hatching to occur within a timeframe of November 29 (AUG1) to May 20 (OCT1) based on our observations (Table 1). These periods of time are still very cold (< 3°C Figure 2), and far from spring warming in our system. In comparison, coho salmon in southcentral systems in Alaska hatch in mid‐April through June and emerge from nests between June and August (Campbell et al. 2019). Beacham and Murray (1990) created emergence estimates for coho embryos and estimated emergence at 344 days, based on average exposure at 1°C and 200 days for 3°C. Furthermore, a summary of emergence approximations outlined by Quinn (2018) suggests 228 days for average exposure of 2°C in coho salmon. Given these emergence estimates, we can approximate that emergence could take place anywhere between March and September for our emulated spawning dates and average temperatures (see Table 1). These approximations applied to our study are highly generalized and as they are based on average thermal exposures but provide a substantiated estimate window for emergence. As such, it would be most beneficial to begin emergence in mid‐June when our stream begins warming above 4°C. This timeframe falls within our experimental spawning dates, which suggests that it is possible to have an environmental emergence match. However, there is evidence to suggest that other interacting factors influence hatching and emergence timeframes in coho salmon in Alaska. Campbell et al. (2019) observed synchronous hatching and emergence in coho salmon populations across the Copper River delta, despite differing spawning dates and rearing conditions. Furthermore, Steel et al. (2012) found evidence to suggest that thermal delivery has significant impacts on emergence timing in Chinook salmon. Taken together, there are clearly other factors that govern hatching and emergence dynamics, especially in wild systems. Nonetheless, our findings support the success of spawning and embryonic development by Arctic establishing individuals.

Our results also further reveal undescribed nuances within a widely used empirical model used to estimate hatch timing in Pacific salmon (Beacham and Murray 1990). The model used to predict days to hatch in our experiment underestimated the number of days to hatch for all spawning days. We found that estimation became increasingly disparate with colder treatments (AUG1 = 14 days, OCT1 = 57 days). Comparable discrepancies have been observed in similar common garden experiments using other Pacific salmon species (e.g., Sparks et al. 2017). Our results suggest that: (1) that these differences may be due to regional differences in populations used to create the predictive model (British Columbia) and our experimental Alaska‐origin salmon (Drenner et al. 2012; Sparks et al. 2017); and (2) that thermal delivery has significant influence on hatching and emergence, such that use of mean temperatures and simple degree day accumulation is insufficient. Care should be taken in identifying thermally suitable streams as determined by thermal accumulation models alone as they do not account for all physiological mechanisms that may facilitate or inhibit developmental matches.

Beyond just temperature, there are many other environmental factors that contribute to embryonic success in the wild that were not captured in our experiment. Our investigation of temperature in a single stream was particularly conservative when considering the complexities of stream temperature heterogeneity that exist and are ever changing in the wild. For instance, Kuhn et al. (2021) demonstrated that physical features, such as system morphology, play a significant role in the patch structuring of thermal refugia as systems warm due to climate change. This is an aspect that may be of particular importance for establishing salmon in a highly hydrologically dynamic Arctic (Prowse et al. 2006). Another paramount factor to successful incubation is substrate quality, which when considered alone can determine interstitial water quality essential for development (e.g., dissolved oxygen levels and other physicochemical factors; Sternecker, Cowley, and Geist 2013; Smialek, Pander, and Geist 2021). Moreover, stressors associated with substrate quality, such as sedimentation, can act synergistically with factors such as temperature to negatively impact incubation and emergence success in salmonids (Sternecker and Geist 2010; Wild, Nagel, and Geist 2023). To add additional nuance, spawning timing likely shapes the level of exposure to these factors and thus has an overarching role in successful (or not) development and emergence (Sternecker, Denic, and Geist 2014). In summary, there is a suite of environmental factors beyond the scope of our study that serve as barriers to success that also must be considered in the investigation of Arctic establishment by Pacific salmon.

The treatments used in this experiment were records of real thermal conditions, which allows us to make some inferences about redistributing subarctic salmon species. Specifically, we can say that current thermal conditions exist in Arctic rivers that can serve as refugia to support survival and incubation of potentially establishing coho salmon. It is important to note that thermal regimes vary across Arctic rivers and suitable conditions for salmon will not be present at all groundwater springs within North Slope Rivers (Dunmall et al. 2016). Implications for spawning success in sites outside of refugia associated with groundwater springs also remains to be understood but may be a barrier to successful incubation and development. Although we are hesitant to put much weight on the survival results, we did observe that survival was possible at these realistic temperatures and were deterministic on the early developmental rate of embryos. More broadly, the results here are consistent with the importance of thermal refugia in the form of groundwater‐fed springs for Arctic establishing Pacific salmon. We highlight a growing need to better understand the holistic impacts that salmon establishment will have on local ecosystems and societies, particularly as the trajectory of more salmon in the Arctic unfolds (Dunmall et al. 2024).

## Author Contributions

**Elizabeth D. Lindley:** conceptualization (equal), data curation (lead), formal analysis (lead), funding acquisition (equal), investigation (lead), methodology (equal), project administration (supporting), writing – original draft (lead). **Karen M. Dunmall:** conceptualization (equal), data curation (equal), formal analysis (supporting), investigation (equal), methodology (equal), resources (equal), software (equal), supervision (equal), validation (equal), writing – review and editing (equal). **Peter A. H. Westley:** conceptualization (equal), data curation (equal), formal analysis (equal), funding acquisition (equal), investigation (equal), methodology (equal), project administration (equal), resources (equal), supervision (lead), validation (equal), writing – review and editing (equal).

## Conflicts of Interest

The authors declare no conflicts of interest.

## Data Availability

Data are archived and freely available on the Knowledge Network for Biocomplexity: https://doi.org/10.5063/F1G73C54.

## References

[ece370797-bib-0001] Armstrong, J. B. , A. H. Fullerton , C. E. Jordan , et al. 2021. “The Importance of Warm Habitat to the Growth Regime of Cold‐Water Fishes.” Nature Climate Change 11: 354–361. 10.1038/s41558-021-00994-y PMC9037341 35475125

[ece370797-bib-0002] Bailey, J. E. , and D. R. Evans . 1971. “The Low‐Temperature Threshold for Pink Salmon Eggs in Relation to a Proposed Hydroelectric Installation.” Fishery Bulletin 69, no. 3: 587–593.

[ece370797-bib-0003] Bates, D. , M. Mächler , B. M. Bolker , and S. C. Walker . 2015. “Fitting Linear Mixed‐Effects Models Using lme4.” Journal of Statistical Software 67, no. 1: 1–48.

[ece370797-bib-0004] Beacham, T. D. , and C. B. Murray . 1987. “Effects of Transferring Pink ( *Oncorhynchus gorbuscha* ) and Chum ( *Oncorhynchus keta* ) Salmon Embryos at Different Developmental Stages to a Low Incubation Temperature.” Canadian Journal of Zoology 65: 96–105.

[ece370797-bib-0005] Beacham, T. D. , and C. B. Murray . 1990. “Temperature, Egg Size, and Eevelopment of Embryos and Alevins of Five Species of Pacific Salmon: A Somparative Analysis.” Transactions of the American Fisheries Society 119, no. 6: 937–945.

[ece370797-bib-0006] Bendock, T. N. 1981. Inventory and Cataloging of Arctic Area Waters. Juneau, Alaska: Alaska Department of Fish and Game, Division of Sport Fish, Federal Aid in Fish Restoration, Study G‐I‐I, Project F‐9‐13.

[ece370797-bib-0007] Bilous, M. , and K. Dunmall . 2020. “Atlantic Salmon in the Canadian Arctic: Potential Dispersal, Establishment, and Interaction With Arctic Char.” Reviews in Fish Biology and Fisheries 30, no. 3: 463–483.

[ece370797-bib-0008] Bolker, B. M. , M. E. Brooks , C. J. Clark , et al. 2009. “Generalized Linear Mixed Models: A Practical Guide for Ecology and Evolution.” Trends in Ecology & Evolution 24, no. 3: 127–135. 19185386 10.1016/j.tree.2008.10.008

[ece370797-bib-0009] Brannon, E. L. 1987. “Mechanisms Stabilizing Salmonid Fry Emergence Timing.” Canadian Special Publication of Fisheries and Aquatic Sciences 96: 120–124.

[ece370797-bib-0010] Brown, C. L. , N. M. Braem , M. L. Kostick , et al. 2016. Harvests and Uses of Wild Resources in 4 Interior Alaska Communities and 3 Arctic Alaska Communities, 2014. Fairbanks, Alaska: Alaska Department of Fish and Game, Division of Subsistence.

[ece370797-bib-0011] Campbell, E. Y. , J. B. Dunham , G. H. Reeves , and S. M. Wondzell . 2019. “Phenology of Hatching, Emergence, and End‐Of‐Season Body Size in Young‐Of‐Year Coho Salmon in Thermally Contrasting Streams Draining the Copper River Delta, Alaska.” Canadian Journal of Fisheries and Aquatic Sciences 76, no. 2: 185–191.

[ece370797-bib-0012] Carothers, C. , T. L. Sformo , S. Cotton , J. C. George , and P. A. H. Westley . 2019. “Pacific Salmon in the Rapidly Changing Arctic: Exploring Local Knowledge and Emerging Fisheries in Utqiaġvik and Nuiqsut, Alaska.” Arctic 72, no. 3: 273–288.

[ece370797-bib-0013] Chila, Z. , K. M. Dunmall , T. A. Proverbs , et al. 2022. “Inuvialuit Knowledge of Pacific Salmon Range Expansion in the Western Canadian Arctic.” Canadian Journal of Fisheries and Aquatic Sciences 79, no. 7: 1042–1055.

[ece370797-bib-0014] Combs, B. D. 1965. “Effect of Temperature on the Development of Salmon Eggs.” Progressive Fish‐Culturist 27, no. 3: 134–137.

[ece370797-bib-0015] Craig, P. , and L. Haldorson . 1986. “Pacific Salmon in the North American Arctic.” Arctic 39, no. 1: 2–7.

[ece370797-bib-0016] Drenner, S. M. , T. D. Clark , C. K. Whitney , E. G. Martins , S. J. Cooke , and S. G. Hinch . 2012. “A Synthesis of Tagging Studies Examining the Behaviour and Survival of Anadromous Salmonids in Marine Environments.” PLoS One 7, no. 3: 1–13. 10.1371/journal.pone.0031311 PMC3303779 22431962

[ece370797-bib-0017] Dunmall, K. M. , J. A. Langan , C. J. Cunningham , et al. 2024. “Pacific Salmon in the Canadian Arctic Highlight a Range‐Expansion Pathway for Sub‐Arctic Fishes.” Global Change Biology 30, no. 6: e17353. 38837850 10.1111/gcb.17353

[ece370797-bib-0018] Dunmall, K. M. , D. G. McNicholl , E. Farley , and J. D. Reist . 2021. “Reported Occurrences of Pacific Salmon in the Canadian Arctic Continue to Increase Whereas Reports of Atlantic Salmon Sightings Remain Low.” North Pacific Anadromous Fish Commission Technical Report 17.

[ece370797-bib-0020] Dunmall, K. M. , D. G. McNicholl , C. E. Zimmerman , S. E. Gilk‐Baumer , S. Burril , and V. R. von Biela . 2022. “First Juvenile Chum Salmon Confirms Successful Reproduction for Pacific Salmon in the North American Arctic.” Canadian Journal of Fisheries and Aquatic Sciences 79, no. 5: 703–707.

[ece370797-bib-0021] Dunmall, K. M. , N. J. Mochnacz , C. E. Zimmerman , C. Lean , and J. D. Reist . 2016. “Using Thermal Limits to Assess Establishment of Fish Dispersing to High‐Latitude and High‐Elevation Watersheds.” Canadian Journal of Fisheries and Aquatic Sciences 73, no. 12: 1750–1758.

[ece370797-bib-0022] Dunmall, K. M. , J. D. Reist , E. C. Carmack , J. A. Babaluk , M. P. Heide‐Jorgensen , and M. F. Docker . 2013. “Pacific Salmon in the Arctic: Harbingers of Change. Responses of Arctic Marine Ecosystems to Climate Change 2012.” In Proceedings for the 28th Lowell Wakefield Fisheries Symposium, edited by F. J. Mueter , D. M. S. Dickson , H. P. Huntington , et al. University of Alaska Fairbanks: Alaska SeaGrant.

[ece370797-bib-0023] Einum, S. , and I. A. Fleming . 2000. “Selection Against Late Emergence and Small Offspring in Atlantic Salmon ( *Salmo salar* ).” Evolution 54, no. 2: 628–639. 10937238 10.1111/j.0014-3820.2000.tb00064.x

[ece370797-bib-0024] Esteve, M. 2005. “Observations of Spawning Behaviour in Salmoninae: *Salmo*, *Oncorhynchus* and *Salvelinus* .” Reviews in Fish Biology and Fisheries 15, no. 1–2: 1–21.

[ece370797-bib-0025] Farley, E. V. , J. M. Murphy , K. Cieciel , et al. 2020. “Response of Pink Salmon to Climate Warming in the Northern Bering Sea.” Deep‐Sea Research Part II: Topical Studies in Oceanography 177, no. 2020: 104830.

[ece370797-bib-0026] Frainer, A. , R. Primicerio , S. Kortsch , et al. 2017. “Climate‐Driven Changes in Functional Biogeography of Arctic Marine Fish Communities.” Proceedings of the National Academy of Sciences of the United States of America 114, no. 46: 12202–12207. 29087943 10.1073/pnas.1706080114 PMC5699037

[ece370797-bib-0027] George, C. , L. Moulton , and M. Johnson . 2009. A Field Guide to the Common Fishes of the North Slope of Alaska. Barrow, Alaska: North Slope Borough.

[ece370797-bib-0028] Giefer, J. , and S. Graziano . 2023. Catalog of Waters Important for Spawning, Rearing, or Migration of Anadromous Fishes—Arctic Region, Effective June 1, 2023. Anchorage, AK: Alaska Department of Fish and Game, Habitat Division.

[ece370797-bib-0050] Hilborn, R. , T. P. Quinn , D. E. Schindler , and D. E. Rogers . 2003. “Biocomplexity and Fisheries Sustainability.” Proceedings of the National Academy of Sciences of the United States of America 100, no. 11: 6564–6568. 10.1073/pnas.1037274100. 12743372 PMC164486

[ece370797-bib-0048] IPCC . 2014. “Climate Change 2014: Synthesis Report.” In Contribution of Working Groups I, II, and III to the Fifth Assessment Report of the Intergovernmental Panel on Climate Change, edited by Core Writing Team , R. K. Pachauri , and L. A. Meyer . Geneva, Switzerland: IPCC.

[ece370797-bib-0029] Kuhn, J. , R. Casas‐Mulet , J. Pander , and J. Geist . 2021. “Assessing Stream Thermal Heterogeneity and Cold‐Water Patches From UAV‐Based Imagery: A Matter of Classification Methods and Metrics.” Remote Sensing 13, no. 7: 1379.

[ece370797-bib-0030] Meisner, J. D. , J. S. Rosenfeld , and H. A. Regier . 1988. “The Role of Groundwater in the Impact of Climate Warming on Stream Salmonines.” Fisheries 13, no. 3: 2–8.

[ece370797-bib-0031] Melbourne‐Thomas, J. , A. Audzijonyte , M. J. Brasier , et al. 2022. “Poleward Bound: Adapting to Climate‐Driven Species Redistribution.” Reviews in Fish Biology and Fisheries 32, no. 1: 231–251. 33814734 10.1007/s11160-021-09641-3 PMC8006506

[ece370797-bib-0032] Mikow, E. , B. Retherford , A. Godduhn , and M. L. Kostick . 2016. Exploring the Subsistence Fisheries of Point Lay and Wainwright. Alaska: Alaska Department of Fish and Game, Division of Subsistence.

[ece370797-bib-0033] Mueter, F. J. , B. Planque , G. L. Hunt , et al. 2021. “Possible Future Scenarios in the Gateways to the Arctic for Subarctic and Arctic Marine Systems: II. Prey Resources, Food Webs, Fish, and Fisheries.” ICES Journal of Marine Science 78, no. 9: 3017–3045.

[ece370797-bib-0034] Murray, C. B. , and T. D. Beacham . 1986. “Effect of Varying Temperature Regimes on the Development of Pink Salmon ( *Oncorhynchus gorbuscha* ) Eggs and Alevins.” Canadian Journal of Zoology 64, no. 3: 670–676.

[ece370797-bib-0035] Nielsen, J. L. , G. T. Ruggerone , and C. E. Zimmerman . 2013. “Adaptive Strategies and Life History Characteristics in a Warming Climate: Salmon in the Arctic?” Environmental Biology of Fishes 96, no. 10–11: 1187–1226.

[ece370797-bib-0036] Pinsky, M. L. , G. Reygondeau , R. Caddell , J. Palacios‐Abrantes , J. Spijkers , and W. W. L. Cheung . 2018. “Preparing Ocean Governance for Species on the Move.” Science 360, no. 6394: 1189–1191. 29903965 10.1126/science.aat2360

[ece370797-bib-0037] Power, G. , R. S. Brown , and J. G. Imhof . 1999. “Groundwater and Fish—Insights From Northern North America.” Hydrological Processes 13, no. 3: 401–422.

[ece370797-bib-0038] Prowse, T. D. , F. J. Wrona , J. D. Reist , et al. 2006. “Climate Change Effects on Hydroecology of Arctic Freshwater Ecosystems.” Ambio 35, no. 7: 347–358. 17256639 10.1579/0044-7447(2006)35[347:cceoho]2.0.co;2

[ece370797-bib-0039] Quinn, T. P. 2018. The Behavior and Ecology of Pacific Salmon and Trout. Seattle: University of Washington press.

[ece370797-bib-0049] R Core Team . 2022. R: A Language and Environment for Statistical Computing. Vienna, Austria: R Foundation for Statistical Computing. https://www.R‐project.org/.

[ece370797-bib-0040] Smialek, N. , J. Pander , and J. Geist . 2021. “Environmental Threats and Conservation Implications for Atlantic Salmon and Brown Trout During Their Critical Freshwater Phases of Spawning, Egg Development and Juvenile Emergence.” Fisheries Management and Ecology 28, no. 5: 437–467.

[ece370797-bib-0041] Sparks, M. M. , P. A. H. Westley , J. A. Falke , and T. P. Quinn . 2017. “Thermal Adaptation and Phenotypic Plasticity in a Warming World: Insights From Common Garden Experiments on Alaskan Sockeye Salmon.” Global Change Biology 23, no. 12: 5203–5217. 28586156 10.1111/gcb.13782

[ece370797-bib-0042] Steel, E. A. , A. Tillotson , D. A. Larsen , A. H. Fullerton , K. P. Denton , and B. R. Beckman . 2012. “Beyond the Mean: The Role of Variability in Predicting Ecological Effects of Stream Temperature on Salmon.” Ecosphere 3, no. 11: 1–11.

[ece370797-bib-0043] Sternecker, K. , D. E. Cowley , and J. Geist . 2013. “Factors Influencing the Success of Salmonid Egg Development in River Substratum.” Ecology of Freshwater Fish 22, no. 2: 322–333.

[ece370797-bib-0044] Sternecker, K. , M. Denic , and J. Geist . 2014. “Timing Matters: Species‐Specific Interactions Between Spawning Time, Substrate Quality, and Recruitment Success in Three Salmonid Species.” Ecology and Evolution 4, no. 13: 2749–2758. 25077024 10.1002/ece3.1128 PMC4113297

[ece370797-bib-0045] Sternecker, K. , and J. Geist . 2010. “The Effects of Stream Substratum Composition on the Emergence of Salmonid Fry.” Ecology of Freshwater Fish 19, no. 4: 537–544.

[ece370797-bib-0046] Tang, J. , M. D. Bryant , and E. L. Brannon . 1987. “Effect of Temperature Extremes on the Mortality and Development Rates of Coho Salmon Embryos and Alevins.” Progressive Fish‐Culturist 49: 167–174.

[ece370797-bib-0051] Wickham, H. 2016. ggplot2: Elegant Graphics for Data Analysis. Springer‐Verlag. https://ggplot2.tidyverse.org.

[ece370797-bib-0047] Wild, R. , C. Nagel , and J. Geist . 2023. “Climate Change Effects on Hatching Success and Embryonic Development of Fish: Assessing Multiple Stressor Responses in a Large‐Scale Mesocosm Study.” Science of the Total Environment 893: 164834. 37327887 10.1016/j.scitotenv.2023.164834

